# Dietary Tributyrin Administration Improves Intestinal Morphology and Selected Bacterial and Short-Chain Fatty Acid Profiles in Broilers Under an Isocaloric Feeding Regime

**DOI:** 10.3389/fmicb.2021.715712

**Published:** 2021-08-04

**Authors:** Qunbing Hu, Fugui Yin, Baocheng Li, Yuming Guo, Yulong Yin

**Affiliations:** ^1^State Key Laboratory of Animal Nutrition, College of Animal Science and Technology, China Agricultural University, Beijing, China; ^2^Institute of Subtropical Agriculture, Chinese Academy of Sciences, Changsha, China; ^3^Hubei Horwath Biotechnology Co., Ltd., Xianning, China

**Keywords:** tributyrin, growth performance, intestinal morphology, intestinal bacterial population, intestinal short-chain fatty acids, broiler

## Abstract

The current study was conducted to investigate the effect of dietary tributyrin (TB) administration on the intestinal and growth performances in Arbor Acres (AA) broilers under an isocaloric feeding regime. A total of 540 day-old healthy AA broilers were randomly assigned to five treatments with 12 replicates (pens) per treatment and nine birds per pen for 42 days. The dietary treatments were basal diet (control) and basal diet with TB at doses of 0.23 g/kg (TB1), 0.46 g/kg (TB2), 0.92 g/kg (TB3), and 1.84 g/kg (TB4). Particularly, to achieve the isocaloric and cost-saving experimental diets, soybean oil was replaced by the TB product (Eucalorie^®^) with equivalent metabolic energy contents, and the formulas were rebalanced with zeolite to get the sum of all the feed ingredients to 100%. On days 21 and 42, after weighing, the birds (one bird per replicate) whose body weight was close to the replicate average were euthanized to investigate the effect of dietary TB on intestinal morphology, intestinal bacterial population, and short-chain fatty acid contents. The results revealed that dietary TB administration increased the average daily gain, gain/feed ratio, and European broiler index (*P* < 0.05) and improved the intestinal morphology (*P* < 0.05) as indicated by higher villus height and the ratios of villus height/crypt depth in broilers. The incremental levels of TB increased the ileal *Lactobacillus* content (*P* = 0.05) and cecal *Bacillus* content (*P* = 0.02), respectively. Moreover, dietary TB administration also increased the contents of most of the selected short-chain fatty acids in ileal and cecal digesta (*P* < 0.05). Collectively, dietary TB administration quadratically improved the growth performance, intestinal morphology, beneficial bacterial population, and short-chain fatty acid levels under the isocaloric feeding regime, indicating better profit return potential in practical poultry operation.

## Introduction

Unlike beef and pork, poultry meat is widely consumed due to its favorable and nutritive value and because it is free from cultural or religious restrictions (Henchion et al., 2014). Thus, poultry production is facing a promising yet challenging future accompanying the increase of global demand for human-edible protein source due to the ever-increasing human population (Wu et al., 2014; Yadav and Jha, 2019). Such demand attracted widespread concerns regarding the health and welfare for optimal growth performance and economic profit return in poultry production.

Intestinal health is of particular importance for the health and welfare of broilers. In animals, the intestinal tract is not only the place for nutrient digestion and absorption but also the largest immune organ for the protection of the body from adverse challenges, such as infectious pathogenic bacteria, virus, and parasites (Sunkara et al., 2011; Kiela and Ghishan, 2016; Citi, 2018); therefore, there is a growing need worldwide to explore strategies able to maintain the integrity of intestinal morphology and modulate the intestinal function. In this regard, butyrate would be a promising choice, as it has been well demonstrated to play fundamental roles in modulating the intestinal structure and microbial composition (Yang et al., 2018; Wang et al., 2019a; Sotira et al., 2020). In addition, aside from its primary function as a preferred energy source over glucose and glutamine for colonic epithelial cells, butyrate is a strong mitosis promoter and a differentiation agent for intestinal epithelial cells (Guilloteau et al., 2010), thus enhancing the intestinal barrier by facilitating tight junction assembly (Peng et al., 2009; Taba et al., 2020). Furthermore, butyrate derivate products could effectively induce the immune response by upregulating the gene expression of intestinal host defense peptides and controlling the proliferation of bacterial pathogens in chickens (Sunkara et al., 2011, 2012). With the above features, butyrate derivate products proved to be a valid aid for gut health maintenance.

Butyrate sodium and tributyrin (TB) are the most commonly available butyrate derivate products; however, their bio-beneficial effects on human and animals are different. Previous clinical and pharmacologic research demonstrated that TB showed advantages over sodium butyrate (Edelman et al., 2003). For example, TB could be easily absorbed to the systemic circulation and increase the butyrate concentration in the portal vein. With its longer metabolic half-life, a sufficient amount of TB could be delivered to the target tissues or organs for the initiation of beneficial effects (Chen and Breitman, 1994; Conley et al., 1998; Miyoshi et al., 2011). Thus, TB has been defined as “a stable and rapidly absorbed prodrug of butyric acid” (Gaschott et al., 2001). Interestingly, the intestinal bacterial composition could be modified in response to different butyrate sources. For example, dietary TB treatment significantly enriched the Bacteroidetes while reducing the Proteobacteria at the phylum level in broilers (Moquet, 2018). Particularly, TB efficiently induces fetal hemoglobin and human cathelicidin expression under physiologically available concentration compared with sodium butyrate, demonstrating that its beneficial bio-efficiency is superior to that of sodium butyrate (Witt et al., 2000; Jiang et al., 2013). Besides, except for acting as an energy source for animals, dietary butyrate treatment improved the apparent metabolizable energy corrected for nitrogen in broilers (Kaczmarek et al., 2016), showing an energy-sparing effect. This is of great economic interest in practical application, because the contents of energy ingredients in the formula could be reduced for cost-saving purposes. The objective of the present study was, therefore, to determine the changes in growth performance, intestinal morphology, and selected bacterial and short-chain fatty acid (SCFA) profiles in response to dietary TB treatment by partially following the isocaloric and cost-saving feed additives-supplementing strategy in broilers.

## Materials and Methods

### Animals, Experimental Design, and Diets

The protocol for the animal experiment was approved by the Animal Care and Use Committee of the China Agricultural University (Ethical experimental statement no. AW13501202-2-1).

A total of 540 day-old healthy Arbor Acres (AA) broiler chickens (half male and half female) were allocated equally on the basis of weight of origin into five dietary treatments in a randomized complete block design. The dietary treatments were the basal diet-fed group (control) and the groups fed with basal diet supplemented with TB, namely, groups TB1 to TB4. The dietary TB levels were 0.23 g/kg (TB1), 0.46 g/kg (TB2), 0.92 g/kg (TB3), and 1.84 g/kg (TB4). There were 12 replicates per treatment with nine birds per replicate (cage). Feed ingredients and feeding program are summarized in Table 1. Particularly, in order to achieve an isocaloric level in each formula, the soybean oil was replaced by the TB product (Eucalorie^®^) with equivalent metabolic energy content, and the formulas were rebalanced with zeolite to get the sum of all the feed ingredients to 100%. The TB product (Eucalorie^®^) contains 92% TB, with a metabolic energy value of 8 MCal/kg for broilers, and was kindly contributed by Hubei Horwath Biotechnology Co., Ltd., China. No antibiotics were used throughout the animal trial.

**TABLE 1:** Ingredient and nutrient composition of the basal diets.

Ingredients (%)	Starter 1–21 days	Finisher 22–42 days	Calculated composition	Starter 1–21 days	Finisher 22–42 days
Corn	56.40	64.32	ME (Mcal/kg)	2.87	2.93
Soybean meal	32.67	25.46	Crude protein (%)	21.58	19.00
Zein (crude protein 90%)	4.00	4.00	Ca (%)	1.00	0.90
Soybean oil	1.39	1.15	Available phosphorus (%)	0.48	0.40
Salt	0.30	0.35	Lys (%)	1.14	1.00
L-Lysine-HCl	0.12	0.17	Met (%)	0.51	0.40
DL-Methionine	0.18	0.10			
Zeolite	1.15	1.15			
Calcium phosphate	2.10	1.64			
Limestone	1.14	1.20			
Antioxidant	0.02	0.03			
Choline chloride (50%)	0.30	0.20			
Mineral premix^a^	0.20	0.20			
Vitamin premix^b^	0.03	0.03			

*^a^Supplied per kilogram of diet: Zn (ZnSO_4_), 75 mg; Fe (FeSO_4_), 80 mg; Mn (MnSO_4_), 100 mg; Cu (CuSO_4_), 8 mg, I (CaI_2_), 0.35 mg; Se (Na_2_SeO_3_), 0.25 mg. ^b^Supplied per kilogram of diet: Vitamin A, 18,750 IU; Vitamin D_3_, 3,750 IU; Vitamin E, 28 IU; Vitamin K_3_, 3.975 mg; Vitamin B_1_, 3 mg; Vitamin B_2_, 9 mg; Vitamin B_5_, 18 mg; Vitamin B_12_, 37.5 μg; biotin, 150 μg; folic acid, 1.875 mg; nicotinic acid, 75 mg.*

The temperature was maintained at 32°C for 5 days and then gradually reduced to 22°C, in keeping with normal brooding practice. A 23 h/day constant-light regime was applied through the entire animal trial. Birds were vaccinated against coccidiosis at 1 day of age following the regular vaccination program. Feed and water were freely available. The body weight was recorded individually prior to feeding in the morning on days 21 and 42, and cumulative feed consumption per cage was recorded at the same time. The dead bird numbers were recorded daily as well. The phase mortality, average daily weight gain (ADG), average daily feed intake (ADFI), feed conversion ratio (FCR), and the European broiler index (EBI) were calculated following a previous method (Kryeziu et al., 2018).

### Intestinal Sections and Digesta Sampling

On days 21 and 42, after being weighed, the birds (one bird per cage) whose body weight was close to the cage average were selected and euthanized for sample collection. Middle duodenal and jejunal, as well as distal ileal, sections (approximately 2 cm in length) were collected followed by removal of digesta and connective adipose tissues, rinsed with pre-cold physiological saline (0.9% saline), and immediately fixed with 4% polyoxymethylene for later intestinal morphological examination. Approximately 2 g of contents from the distal ileum and cecum was collected and aseptically emptied in 5-ml sterile tubes for later bacterial enumeration. The remaining digesta in the distal ileum and cecum were collected in 50-ml sterile tubes, immediately frozen with liquid nitrogen, and stored at −80°C until the later analysis of volatile fatty acid (VFA) concentrations.

### Intestinal Histomorphology Analysis

To evaluate the intestinal histomorphology, selected intestinal tissues were fixed with 4% polyoxymethylene for 24 h and then embedded in petrolin and sectioned into 2–3-μm sections. The sections were stained with hematoxylin first, counterstained with eosin, and finally observed under an optical microscope following previous procedures (Wang et al., 2018). The villus height (the distance from the apex of the villus to the junction of the villus and crypt) and crypt depth (the distance from the junction to the basement membrane of the epithelial cells at the bottom of the crypt) were measured on 10 intact, well-oriented villi per intestinal section, and then the ratio of villus height/crypt depth was calculated.

### Intestinal Bacterial Quantitation

Conventional agar plating was used for enumerating viable bacteria. The selected intestinal bacterial quantitation, including *Bacillus*, *Lactobacillus*, and *Coliforms*, was performed following previous procedures with minor modifications (Proietti et al., 2009; Hu et al., 2012; Yin et al., 2014). Briefly, 0.5 g of fresh intestinal content was plated directly on appropriate selective agar media, in duplicate, by Eddy Jet Spiral Plater (Neutec Group Inc., Farmingdale, NY, United States) after a series of 10-fold dilution in physiological saline supplemented with 0.1% peptone with a detection limit of 100 CFU/g digesta. The enumeration of *Bacillus* was assayed on tryptone soy agar after aerobic incubation at 37°C for 24 h, that of *Lactobacillus* was determined on de Man, Rogosa, and Sharpe agar anaerobically incubated at 37°C for 48 h, and that of *Coliform* bacteria was enumerated on MacConkey agar following aerobic incubation at 37°C for 24 h. The bacterial enumeration was expressed in colony-forming units (log10 CFU/g of fresh digesta).

### Intestinal SCFA Measurement

The concentrations of SCFAs, including acetic acid, propionic acid, isobutyric acid, butyric acid, isovaleric acid, valeric acid, and total VFAs, in the digesta of selected intestinal sections were determined according to previous procedures with minor modifications (Park and Park, 2014; Park and Kim, 2017). Briefly, 2 g of digesta was placed in a screw-cap tube and mixed with 4 ml of ultra-pure water and then homogenized and centrifuged at 4,000 rpm for 10 min at 4°C. Approximately 1 ml of the supernatant was aliquoted into another ampule and acidified by adding 0.2 ml of 25% HPO_3_ solution, mixed well and put on an ice bath for 3 h, and then centrifuged at 10,000 × *g* for 10 min at 4°C, and 1.0 μl of the supernatant was collected and injected into a gas chromatographic system (Model GC-15A, Shimadzu Corp., Kyoto, Japan), equipped with a column (Supelco, Inc., Bellefonte, PA, United States) filled with 10% SP-1000 and a flame ionization detector. The initial temperatures of the oven, injector, and flame ionization detector were 100, 240, and 250°C, respectively. The column was operated at 100–150°C, along with highly purified N_2_ (1.8 ml/min) as a carrier gas. The results were expressed as μmol/g digesta.

### Statistical Analysis

The data were analyzed using the general linear model procedure of the SPSS program (version 17.0) for analysis of variance. The nature of the response to increasing concentration of dietary TB was determined by polynomial contrasts. The model included linear and quadratic contrasts for effects of dietary TB. The multiple comparisons were tested by the Tukey–Kramer method to determine differences among treatment means. A *P*-value < 0.05 was taken to indicate statistical significance between treatments.

## Results

### Growth Performance

The FCR in TB3 and TB4 groups were lower than that in the other three groups (*P* < 0.05), although no significant differences of ADG and ADFI were observed among treatments (*P* > 0.05) in the first feeding period (0–21 days). The ADG and ADFI in the second (22–42 days) and overall (0–42 days) feeding periods as well as the FCR in the second feeding period were quadratically improved in response to dietary TB administration (*P* < 0.05. Particularly, the ADG and ADFI were highest in the TB4 group (*P* < 0.05), while the FCR was lowest mainly in the TB3 and TB4 groups (*P* < 0.05) in these feeding periods. The results are shown in Table 2.

**TABLE 2:** Effect of dietary TB on growth performance in broilers.

Item	Control	TB1	TB2	TB3	TB4	Pooled SEM	Treatment *P*-value	Liner *P*-value	Quadratic *P*-value
**0–21 days**									
ADG (g)	26.25	26.26	26.99	27.98	28.46	0.04	0.11	0.11	0.67
ADFI (g)	41.71	41.92	42.78	43.47	44.01	0.04	0.34	0.06	0.96
FCR	1.59^a^	1.60^a^	1.59^a^	1.55^b^	1.55^b^	0.00	0.03	0.85	0.50
Mortality (%)	2.78	1.85	2.78	1.85	1.85	–	–	–	–
EBI	160.50	161.09	162.98	179.49	180.22	–	–	–	–
**22–42 days**									
ADG/g	72.94^b^	74.06^ab^	74.34^ab^	75.07^ab^	81.41^a^	0.15	0.02	0.07	0.02
ADFI/g	134.14^b^	136.13^b^	136.51^b^	136.70^b^	146.48^a^	0.21	0.04	0.04	0.05
FCR	1.84^a^	1.84^b^	1.84^ab^	1.82^b^	1.80^b^	0.00	0.04	0.05	0.05
Mortality (%)	5.32	3.23	3.19	2.15	2.13	–	–	–	–
EBI	369.30	399.66	389.66	403.60	442.64	–	–	–	–
**0–42 days**									
ADG/g	47.74^b^	48.74^b^	49.22^ab^	50.20^ab^	53.86^a^	0.12	0.05	0.02	0.01
ADFI/g	85.89^b^	85.28^b^	87.51^ab^	87.36^ab^	93.35^a^	0.20	0.03	0.10	0.05
FCR	1.80^a^	1.75^ab^	1.78^ab^	1.74^b^	1.73^b^	0.00	0.04	0.38	0.31
Mortality (%)	6.33	5.38	4.48	4.55	3.55	–	–	–	–
EBI	248.43	263.53	264.13	275.38	300.28	–	–	–	–

*ADFI, average daily feed intake; ADG, average daily gain; EBI, European broiler index; FCR, feed conversion ratio; SEM, standard error of the mean; TB, tributyrin. Control = basal diet; TB1 = basal diet supplemented with 230 mg/kg TB; TB2 = basal diet supplemented with 460 mg/kg TB; TB3 = basal diet supplemented with 920 mg/kg TB; TB4 = basal diet supplemented with 1,840 mg/kg TB. ^a,b^Mean values within a row with unlike superscript letters were significantly different (P < 0.05).*

### Histomorphology of Small Intestinal Sections

Dietary TB treatment ameliorated the duodenal and jejunal villus length, crypt depth, and villus length/crypt depth ratio at both 21 and 42 days of ages (*P* < 0.05). The ileal villus length at 21 days of age and the ileal crypt depth at 42 days of age in the TB treatment groups were improved when compared with those in the control group (*P* < 0.05). Particularly, the jejunal villus length; duodenal and jejunal crypt depth; duodenal, jejunal, and ileal villus length/crypt depth ratios at 21 days of age; and the duodenal villus length and villus length/crypt depth ratio at 42 days of age were quadratically changed in association with the dietary TB administration (*P* < 0.05), with the most effective being observed in the TB3 and TB4 groups. The results are shown in Table 3.

**TABLE 3:** Effect of dietary TB on histomorphology of the small intestinal sections in broilers.

Item	Intestine	Control	TB1	TB2	TB3	TB4	Pooled SEM	Treatment *P*-value	Liner *P*-value	Quadratic *P*-value
**21 days**										
Duodenum	Villus length (μm)	1,328.2^b^	1,422.4^b^	1,452.9^ab^	1,456.1^ab^	1,477.9^a^	6.86	0.05	0.42	0.16
	Crypt depth (μm)	414.0^a^	400.6^ab^	380.5^ab^	365.0^bc^	330.9^c^	3.77	0.00	0.03	0.01
	Villus length/crypt depth	3.21^c^	3.55^bc^	3.82^b^	3.99^ab^	4.47^a^	0.06	0.00	0.07	0.02
Jejunum	Villus length (μm)	926.8^c^	967.5^bc^	1,019.3^ab^	1,056.7^a^	1,071.4^a^	7.07	0.00	0.22	0.01
	Crypt depth (μm)	304.8^a^	295.00^ab^	291.0^ab^	276.7^b^	261.7^b^	1.96	0.06	0.03	0.00
	Villus length/crypt depth	3.04^c^	3.28^b^	3.50^ab^	3.82^ab^	4.09^a^	0.05	0.00	0.07	0.00
Ileum	Villus length (μm)	659.2^c^	746.1^b^	758.7^b^	794.1^a^	804.4^a^	6.69	0.00	0.35	0.08
	Crypt depth (μm)	196.5	194.0	183.5	179.7	178.3	0.97	0.64	0.28	0.06
	Villus length/crypt depth	3.35^b^	3.85^ab^	4.13^ab^	4.42^a^	4.51^a^	0.05	0.01	0.27	0.02
**42 days**										
Duodenum	Villus length (μm)	1,456.0^c^	1,503.4^bc^	1,522.7^b^	1,545.1^ab^	1,614.6^a^	6.79	0.01	0.05	0.03
	Crypt depth (μm)	401.1^a^	373.6^b^	359.3^b^	368.7^b^	353.9^b^	2.14	0.01	0.45	0.29
	Villus length/crypt depth	3.63^b^	4.02^ab^	4.24^ab^	4.19^ab^	4.56^a^	0.04	0.00	0.21	<0.01
Jejunum	Villus length (μm)	926.8^c^	1,098.4^b^	1,127.7^ab^	1,119.5^ab^	1,170.3^a^	10.96	0.00	0.47	0.25
	Crypt depth (μm)	313.2^a^	291.8^ab^	287.9^b^	285.7^b^	275.1^b^	1.63	0.02	0.72	0.40
	Villus length/crypt depth	2.96^b^	3.76^a^	3.92^a^	3.92^a^	4.25^a^	0.05	0.00	0.43	0.30
Ileum	Villus length (μm)	863.6	870.5	894.5	899.2	899.9	2.32	0.72	0.59	0.15
	Crypt depth (μm)	221.0^a^	204.2^b^	200.4^b^	200.2^b^	198.9^b^	1.33	0.03	0.69	0.49
	Villus length/crypt depth	3.91^b^	4.25^a^	4.46^a^	4.49^a^	4.52^a^	0.03	0.02	0.59	0.30

*SEM, standard error of the mean; TB, tributyrin. Control = basal diet; TB1 = basal diet supplemented with 230 mg/kg TB; TB2 = basal diet supplemented with 460 mg/kg TB; TB3 = basal diet supplemented with 920 mg/kg TB; TB4 = basal diet supplemented with 1,840 mg/kg TB. ^a,b,c^Mean values within a row with unlike superscript letters were significantly different (P < 0.05).*

### Bacterial Populations in Ileal and Cecal Digesta

Dietary TB treatment significantly (*P* < 0.05) affected the selected bacterial (*Bacillus*, *Lactobacillus*, and *Coliforms*) counts in ileal and cecal digesta (Table 4). Specifically, the ileal *Bacillus* and *Lactobacillus* counts and cecal *Coliforms* counts at both 21 and 42 days of age, the cecal *Lactobacillus* counts at 21 days of age, and the cecal *Bacillus* counts at 42 days of age were quadratically changed in response to the dietary TB administration (*P* < 0.05), with the most affected being mainly in the TB3 and TB4 groups.

**TABLE 4:** Effect of dietary TB on ileal and cecal bacterial populations (log10 CFU/g fresh digesta) in broilers.

Item	Bacteria	Control	TB1	TB2	TB3	TB4	Pooled SEM	Treatment *P*-value	Liner *P*-value	Quadratic *P*-value
**21 days**										
Ileal digesta	*Bacillus*	4.76^b^	4.89^b^	5.28^a^	5.35^a^	5.43^a^	0.04	0.00	0.30	0.07
	*Lactobacillus*	4.53^c^	5.19^b^	5.24^b^	5.62^b^	6.35^a^	0.08	0.00	0.06	0.05
Cecal digesta	*Bacillus*	6.62^b^	6.71^b^	6.77^b^	7.33^a^	7.54^a^	0.05	0.00	0.44	0.34
	*Lactobacillus*	6.87^b^	6.96^ab^	7.10^ab^	7.15^ab^	7.36^a^	0.02	0.00	0.61	0.06
	*Coliforms*	7.49^a^	6.90^b^	6.61^b^	6.46^b^	6.47^b^	0.05	0.05	0.43	0.07
**42 days**										
Ileal digesta	*Bacillus*	3.95^b^	4.16^ab^	4.25^ab^	4.41^ab^	4.74^a^	0.03	0.04	0.19	0.19
	*Lactobacillus*	4.03^c^	4.18^bc^	4.51^ab^	4.71^ab^	4.96^a^	0.04	0.00	0.10	0.02
Cecal digesta	*Bacillus*	6.65	6.75	6.90	6.98	6.91	0.02	0.00	0.52	0.02
	*Lactobacillus*	5.93^b^	6.15^b^	6.20^ab^	6.60^a^	6.63^a^	0.04	0.00	0.27	0.10
	*Coliforms*	7.14^a^	7.00^a^	6.76^ab^	6.50^b^	6.32^b^	0.04	0.01	0.82	0.07

*SEM, standard error of the mean; TB, tributyrin. Control = basal diet; TB1 = basal diet supplemented with 230 mg/kg TB; TB2 = basal diet supplemented with 460 mg/kg TB; TB3 = basal diet supplemented with 920 mg/kg TB; TB4 = basal diet supplemented with 1,840 mg/kg TB. ^a,b,c^Mean values within a row with unlike superscript letters were significantly different (P < 0.05).*

### SCFA Concentrations in Ileal and Cecal Digesta

Dietary TB treatment significantly affected the ileal acetic acid content at 21 days of age (*P* < 0.05). The ileal propionic acid content at both 21 and 42 days of age and the cecal contents of all the selected SCFAs at both 21 and 42 days of age were improved in response to dietary TB treatment when compared with those in the control group (*P* < 0.05). Notably, the ileal acetic acid content at 21 days of age, ileal propionic acid content, and cecal propionic acid, butyric acid, and isovaleric acid contents at both 21 and 42 days of age, as well as the cecal acetic acid, isobutyric acid, valeric acid, and total cecal VFAs at 42 days of age were changed quadratically in response to the dietary TB administration (*P* < 0.05), with the most effective being in the TB4 group. The results are shown in Table 5.

**TABLE 5:** Effect of dietary TB on SCFA concentration (μmol/g fresh digesta) in ileal and cecal digesta in broilers.

Item	Bacteria	Control	TB1	TB2	TB3	TB4	Pooled SEM	Treatment *P*-value	Linear *P*-value	Quadratic *P*-value
**21 days**										
Ileal digesta	Acetic acid	6.81^b^	7.19^b^	7.39^ab^	7.52^a^	7.98^a^	0.05	0.00	0.08	0.04
	Propionic acid	0.81^b^	0.84^b^	0.88^b^	0.89^b^	1.01^a^	0.01	0.00	0.03	0.03
Cecal digesta	Acetic acid	42.87^b^	54.92^a^	56.29^a^	57.68^a^	59.74^a^	0.77	0.00	0.41	0.27
	Propionic acid	1.87^c^	2.04^bc^	2.48^ab^	2.69^a^	2.73^a^	0.05	0.01	0.28	0.03
	Isobutyric acid	0.36^b^	0.45^b^	0.50^ab^	0.51^ab^	0.59^a^	0.01	0.01	0.16	0.08
	Butyric acid	11.27^b^	12.11^b^	12.20^b^	14.07^ab^	15.20^a^	0.17	0.02	0.05	0.03
	Isovaleric acid	0.16^c^	0.33^b^	0.39^b^	0.47^b^	0.52^a^	0.02	0.00	0.24	0.04
	Valeric acid	0.99^b^	1.01^b^	1.19^ab^	1.32^a^	1.34^a^	0.02	0.00	0.23	0.06
	Total VFAs	65.14^b^	78.89^b^	81.32^a^	85.15^a^	89.11^a^	1.04	0.00	0.33	0.18
**42 days**										
Ileal digesta	Acetic acid	8.09	8.42	8.64	8.81	8.98	0.04	0.22	0.49	0.21
	Propionic acid	0.76^b^	0.81^b^	0.84^b^	0.89^ab^	0.97^a^	0.02	0.00	0.03	0.00
Cecal digesta	Acetic acid	42.93^b^	44.49^b^	45.99^ab^	48.25^ab^	52.31^a^	0.43	0.03	0.01	0.00
	Propionic acid	3.65^b^	3.81^ab^	3.85^ab^	3.96^ab^	4.22^a^	0.02	0.01	0.03	0.02
	Isobutyric acid	0.76^b^	0.81^b^	0.87^ab^	0.98^ab^	1.06^a^	0.01	0.04	0.05	0.00
	Butyric acid	11.42^c^	12.20^c^	13.43^bc^	15.06^ab^	15.94^a^	0.22	0.01	0.10	0.01
	Isovaleric acid	0.86^b^	0.93^b^	0.98^b^	1.18^ab^	1.36^a^	0.02	0.02	0.02	0.01
	Valeric acid	1.61^b^	1.63^b^	1.70^ab^	1.74^ab^	1.83^a^	0.01	0.04	0.01	0.02
	Total VFAs	70.08^b^	73.10^b^	76.30^ab^	80.87^a^	86.67^a^	0.76	0.03	0.01	0.04

*SEM, standard error of the mean; TB, tributyrin. Control = basal diet; TB1 = basal diet supplemented with 230 mg/kg TB; TB2 = basal diet supplemented with 460 mg/kg TB; TB3 = basal diet supplemented with 920 mg/kg TB; TB4 = basal diet supplemented with 1,840 mg/kg TB. ^a,b,c^Mean values within a row with unlike superscript letters were significantly different (P < 0.05).*

## Discussion

Glyceryl butyrate has been demonstrated to beneficially improve the growth performance and carcass yield in broiler chickens (Leeson et al., 2005; Mansoub et al., 2011; Yin et al., 2016). Such effect is superior to that of probiotics and prebiotics as well as their combination (Taherpour et al., 2009), which have brought great success in the industrial poultry operation. However, with the inevitable yet sustained commercial competition, research scientists and technical experts are keen to explore novel strategies to squeeze more beneficial returns in poultry operation. One promising strategy is to supplement optimal doses of butyrate to the feed formula without increasing the feed cost burden. This is particularly challenging as solid scientific proofs are needed. It is of great interest that dietary butyrate intervention improved the apparent metabolizable energy (Kaczmarek et al., 2016), ileal energy digestible coefficient (Liu et al., 2017), and apparent ileal digestibility of methionine (Moquet et al., 2018) in broilers. In addition, the present study also showed promising results as the ADG and EBI were improved following optimal dietary TB administration at 1,840 mg/kg under the cost-saving formulating regime. Furthermore, several aggressive yet pioneered research showed that better weight gain and feed/gain ratio could be achieved when birds were fed diet with reduced crude protein and/or metabolic energy contents and supplemented with butyrate products (M’Sadeq et al., 2016; Bortoluzzi et al., 2017; Petrilla et al., 2018; Mátis et al., 2019). Taken together, these results provided scientific proofs regarding the application of butyrate for cost-saving and better profit return formulas in poultry operation.

The gastrointestinal tract presents a heavy burden for the digestive and absorptive functions as well as disease resistance ability in animals (Mitchell and Moretó, 2006; Timbermont et al., 2010; Wasti et al., 2020). The improvement of intestinal villi height and villus length/crypt depth ratio is directly linked to better disease prevention, nutrient absorption, and growth performance in animals (Sen et al., 2012). In the present study, the intestinal villi height and villus length/crypt depth ratios were effectively improved in the TB3 and TB4 groups at both 21 and 42 days, demonstrating better morphological and functional improvement of the small intestine under the current TB administration regime. This is of significant importance for broiler production as it ensures that birds achieve a heavier marketing weight in a given time period. Interestingly, higher intestinal villi length and villus length/crypt depth ratios were correlated with higher intestinal weight and longer intestinal length in response to butyrate product administration in broilers (Gong et al., 2011; Lan et al., 2020). Particularly, a heavier gastrointestinal tract was observed in the TB-fed broilers when compared with that in the butyric acid- and sodium butyrate-fed ones (Moquet, 2018), indicating that TB is superior to butyric acid and sodium butyrate in stimulating the growth and development of the gastrointestinal tract in broilers. Besides, TB also effectively protected the intestine from lethal infection-caused hemorrhage and necrosis (Gu et al., 2017; Onrust et al., 2020) and severe oxidative stress-induced damages (Wang et al., 2019b) in broiler and pigs. Collectively, these scientific evidences well supported the improvement of growth performance and well-being in the TB-fed birds.

Gut microbiota is a strong determinant of host physiology and health status; particularly, it plays a critical role in maintaining the normal physiological function of the intestine (Sekirov et al., 2010; Le Chatelier et al., 2013). Previous research has revealed that dietary butyrate treatment beneficially reduced some specific pathogenic bacterial contents, such as *Clostridium perfringens* and *Salmonella enteritidis*, in broilers (Shojadoost et al., 2012; Onrust et al., 2020). Furthermore, a previous study with pyrosequencing techniques revealed that dietary glyceryl butyrate effectively reduced the abundance of pathogenic *Mollicutes* and *Holdemania* in the cecum of broilers (Yang et al., 2018). Because many *Mollicutes* members cause diseases (such as colitis) in humans and animals (Brown et al., 2010), while *Holdemania* has also been reported to be associated with unhealthy ceca in animals (Bailey, 2013), the reduction in the abundance of *Mollicutes* and *Holdemania* with glyceryl butyrate treatment demonstrated its beneficial effects on chicken intestinal health. Interestingly, the current results were in agreement with these findings as dietary TB administration improved the beneficial *Bacillus* and *Lactobacillus* contents while reducing the pathogenic *Coliforms* contents in the ileum and cecum and thus secured better health and well-being in broilers.

In the present study, the cecal concentrations of SCFAs (mainly acetic acid, propionic acid, and butyric acid) increased markedly regardless of the age in days, with higher doses of TB treatment being the most effective. Similar results were observed with higher doses of sodium butyrate treatment in broilers (Wu et al., 2018). However, differences also existed regarding the changes of these SCFA contents in response to different levels of dietary TB and/or sodium butyrate treatment. Particularly, the cecal acetic acid, propionic acid, and butyric acid contents were increased steadily with the increase of dietary TB levels. This is probably because the TB could be stably and rapidly absorbed to the blood circulation (Gaschott et al., 2001) and delivered to the whole gastrointestinal tissues through the arterial blood stream (Edelman et al., 2003; Miyoshi et al., 2011). With its longer metabolic half-life, TB could effectively stimulate the synthesis of host defense peptides in gastrointestinal cells and stimulate the proliferation of beneficial bacteria as well as prohibit that of pathogenic ones (Cuperus et al., 2013; Robinson et al., 2015; 2018). In addition, the increase of intestinal SCFA concentrations is associated with lower intestinal pH value (Meimandipour et al., 2011), which is also beneficial for the growth of intestinal probiotic bacteria in broilers. Besides, butyrate and/or propionate presence in the ceca and colon resulted in significantly heavier jejuna and longer small intestinal and total tract retention times (Moquet et al., 2018); therefore, the higher intestinal SCFA concentrations were positively correlated with the improvement of intestinal histomorphology and growth performance in the current study.

In summary, the present study has shown that dietary TB administration significantly improved the growth performance, intestinal morphology, beneficial bacterial population, and SCFA levels in a dose-dependent quadratic manner under an isocaloric and cost-saving feeding program. These findings provide a solid scientific reference for better economic profit return potential in practical poultry operation.

## Data Availability Statement

The original contributions presented in the study are included in the article/supplementary material, further inquiries can be directed to the corresponding author/s.

## Ethics Statement

The animal study was reviewed and approved by China Agricultural University Laboratory Animal Welfare and Animal Experimental Ethical Committee.

## Author Contributions

QH designed the study. QH and BL conducted the animal trial and sample and data collection. QH and FY analyzed the data and drafted the manuscript. YG and YY reviewed the manuscript. All authors approved the final version of the manuscript.

## Conflict of Interest

QH, FY, and BL were employed by company: Hubei Horwath Biotechnology Co., Ltd. The remaining authors declare that the research was conducted in the absence of any commercial or financial relationships that could be construed as a potential conflict of interest.

## Publisher’s Note

All claims expressed in this article are solely those of the authors and do not necessarily represent those of their affiliated organizations, or those of the publisher, the editors and the reviewers. Any product that may be evaluated in this article, or claim that may be made by its manufacturer, is not guaranteed or endorsed by the publisher.
